# Long-term impact of changing childhood malnutrition on rotavirus diarrhoea: Two decades of adjusted association with climate and socio-demographic factors from urban Bangladesh

**DOI:** 10.1371/journal.pone.0179418

**Published:** 2017-09-06

**Authors:** Sumon Kumar Das, Mohammod Jobayer Chisti, Mohammad Habibur Rahman Sarker, Jui Das, Shawnawaz Ahmed, K. M. Shahunja, Shamsun Nahar, Nora Gibbons, Tahmeed Ahmed, Abu Syed Golam Faruque, Mustafizur Rahman, George J Fuchs, Abdullah Al Mamun, Peter John Baker

**Affiliations:** 1 International Centre for Diarrhoeal Disease Research, Bangladesh (icddr,b), Dhaka, Bangladesh; 2 Institute for Social Science Research, The University of Queensland, Brisbane, Australia; 3 Jahangirnagar University, Savar, Dhaka, Bangladesh; 4 University of Arkansas for Medical Sciences, Little Rock, Arkansas, United States of America; 5 University of Kentucky College of Medicine, Lexington, United States of America; 6 School of Public Health, The University of Queensland, Brisbane, Australia; Universidade de Sao Paulo, BRAZIL

## Abstract

**Background:**

There is strong association between childhood rotavirus, diarrhoea, climate factors and malnutrition. Conversely, a significant nutritional transition (reduced under-nutrition) with a concurrent increasing trend of rotavirus infection in last decade was also observed among under 5 children, especially in developing countries including Bangladesh. Considering the pathophysiology of rotavirus, there might be an interaction of this nutrition transition which plays a pivotal role in increasing rotavirus infection in addition to climate and other man-made factors in urban areas such as Dhaka, Bangladesh.

**Methods:**

Relevant monthly data from 1993–2012 were extracted from the archive of the Diarrhoeal Disease Surveillance System of icddr, b and linked with data collected from the Dhaka station of the Bangladesh Meteorological Department (mean temperature, rainfall, sea level pressure and humidity). Seasonal autoregressive integrated moving average time series models were deployed to determine the association between the monthly proportion of rotavirus infection and underweight, stunting and wasting adjusting for climate, socio-demographic and sanitation factors.

**Finding:**

The proportion of rotavirus cases among all causes diarrhoea increased from 20% in 1993 to 43% in 2012 (Chi squared for trend p = 0.010). In contrast, underweight, stunting and wasting decreased from 59%-29% (p<0.001); 53%-21% (p<0.001) and 32%-22% (p<0.001) respectively over the same period. Mean ambient temperature increased from 25.76°C-26.62°C (p = 0.07); mean rainfall, sea level pressure and mean humidity decreased from 234.92–111.75 mm (p = 0.5), 1008.30–1006.61 mm of hg (p = 0.02) and 76.63%-70.26% (p<0.001), respectively. In the adjusted model, a decrease in monthly proportion of underweight [coef.: -0.189 (95% CI:-0.376, -0.003)] and wasting [-0.265 (-0.455, -0.075)] were significantly and inversely associated with rotavirus infection. However, an inverse but insignificant association was observed for stunting [-0.070 (-0.249, 0.109)].

**Interpretation:**

The reduction of acute childhood malnutrition is significantly associated with increasing rotavirus diarrhoea among under-5 children. Thus mass vaccination in addition to interventions directed at man-made modifiable predictors for prevention and control is warranted.

## Introduction

Childhood diarrhoea remains a major concern in developing Bangladesh [1]. There is a strong association between childhood diarrhoeal disease and several climate factors. For example, an increasing number of diarrhoea cases due to *Vibrio cholera* has been observed with both high and low rainfall [2], while rising temperature as well as high and low rainfall both predict increasing non-cholera cases [3]. These might be closely related to raising of river levels [3] resulting in flooding that effects the most vulnerable groups in poor socio-economic areas and where water-satiation practices are are suboptimal [4]. Flooding is also associated with rapid land coverage (loss of arable land, habitat destruction and the decline in natural vegetation cover) in the Dhaka megacity [5–7]. Moreover, overcrowding, poor sewer systems and waste disposal with water stagnantion, increasing surface temperature and environmental pollution together [7] and concurrently facilitates other infections including typhoid and dengue fever in Dhaka [8–10].

Epidemiological studies suggested a strong association between childhood malnutrition and increased risk of infectious diarrhoea [11]. Among all causes of childhood diarrhoea, rotavirus is one of the most significant attributable pathogens [1] and cellular attachment with healthy cells in the brush border of the intestine is fundamental in the pathophysiology of the rotavirus infection [12–14]. As a result and, compared to undernourished children, rotavirus infection disproportionately affects well-nourished children [15,16].

Global warming with climate change effects ecosystems and might also predict emergence and re-emergence of various infections [17–19] [20]. Data from the long established diarrhoeal disease surveillance system (DDSS) [21] of the Dhaka Hospital at International Centre for Diarrhoeal Disease Research, Bangladesh (icddr,b) observed an association of an increased incidence of rotavirus diarrhoea with high temperature, low humidity and high river-levels [22] with seasonal variability [23, 24]. The facility also experienced a sustained rising trend of rotavirus infection over the last two decades [25] despite a significant improvement of water and sanitation practices in the capital city and its neighbouring catchment areas [24]. Similar to other countries, the profile of childhood malnutrition in Bangladesh has shifted over time with a more recent decrease in under nutrition and increase in over nutrition [24]. Thus, there might be an individual or combined contribution of changing climate factors and decrease in childhood under nutrition to the rising trend of rotavirus infection among children less than 5 years old.

The DDSS has maintained an around the clock diarrhoeal disease surveillance system for the last three decades [15,21,24]. This surveillance system prospectively collects information on diarrhoeal patients and the aetiology, including rotavirus. We have linked this data with different meteorological indicators such as temperature, rainfall, sea level pressure and humidity over the last two decades (1993 to 2012). This enables determination of the long term relationship of the changing pattern of childhood malnutrition, meteorological indicators, and other socio-demographic and sanitation factors with rotavirus diarrhoea in children less than 5 years of age in urban Dhaka, Bangladesh.

## Materials and methods

### Study site and diarrhoeal disease surveillance system

The Dhaka Hospital of icddr,b is located in Dhaka, the capital city of Bangladesh. Since 1962, icddr,b has operated this large urban diarrhoeal disease facility which currently provides care and treatment to approximately 140,000 patients of all ages each year. Most patients are residents of urban and peri-urban Dhaka and the majority are of poor socio-economic background. In addition to patient care services, clinical research on enteric diseases, other infectious diseases and non-communicable diseases is also conducted. The DDSS of the Dhaka Hospital was established in 1979 to study a systematic 4% sample until 1995, when the increase in patient number enable the change to a 2% sample beginning in 1996 of all patients attending this facility irrespective of age, sex, disease severity or socioeconomic context (details are described elsewhere [21]). For the DDSS and following standard methods, a fresh stool spaciemen is collected from each enrolled patient and tested for rotavirus, *Vibrio cholerae*, enterotoxigenic *Escherichia coli* (ETEC) and *Shigella* spp. following standard laboratory methods, reported elsewhere in detail [26–28]. Relevant data for the last two decades (1993–2012) was extracted from the electronic database of the DDSS for analysis.

### Meteorological data

Data on daily maximum and minimum temperature, rainfall, sea level pressure and relative humidity were obtained from the Dhaka station of the Bangladesh Meteorological Department [22]. Meteorological data for months and years were determined from the daily records.

### Assessment of nutritional status and malnutrition

Anthropometric measurements of children (weight and length/height) were measured [weight was measured to the nearest 100 gm using a digital scale, length/height was measured using a locally constructed length board or stadiometer with a precision of 0.1 cm] by trained and experienced Research Assistants following standard procedures [29]. All measurements were compared to the WHO 2006 growth standards and the nutritional status defined by z-score underweight (weight-for-age z score < -2 sd), stunting (height-for-age z score < -2 sd), and wasting (weight-for-height z score < -2 sd) [29].

### Socio-demographic factors

Monthly mean age and proportion of females under 5 children enrolled in the DSSS, monthly proportion of non-sanitary toilet uses, non-slum residence, greater than one under 5 year children in the household were estimated and included in the analyses with the aim to determine the adjusted association.

### Ethical considerations

The Diarrhoeal Disease Surveillance System (DDSS) of icddr,b is an established ongoing activity of the Dhaka Hospital approved by the Research Review Committee (RRC) and Ethical Review Committee (ERC) of icddr,b. At enrollment, verbal consent is taken from the caregivers or guardians on behalf of the patients following the hospital policy. This verbal consent was documented by keeping a check mark in the questionnaire which was again shown to the patient or the parents. Parents or guardians were assured about the non-disclosure of information collected from them, and were also informed about the use of data for analysis and using the results for improving patient care activities, conducting researches as well as publication without disclosing the name or identity of their children. ERC was satisfied with the voluntary participation, maintenance of the rights of the participants and confidential handling of personal information by the hospital physicians and has approved this consent procedure.

### Data analysis

All relevant data in the DDSS and meteorological indicators were collected in daily basis. From these daily data, the monthly proportion of rotavirus infection, proportion of malnutrition (underweight, stunting and wasting) among children under 5 years, monthly mean ambient temperature, rainfall, sea level pressure and humidity with other socio-demographic factors were calculated. A time series analysis was performed to explore the relationship between the monthly proportion of rotavirus infection with the monthly proportion of malnutrition (the main exposure), monthly mean ambient temperature, rainfall, sea level pressure and humidity using a seasonal autoregressive integrated moving average (ARIMA) model [30,31]. Firstly, the autocorrelation function (ACF; S1 Fig) and partial autocorrelation function (PACF; S2 Fig) plots of the differenced series were used to identify the number of autoregressive and/or moving average (MA) terms for inclusion in the model. Cross-correlations to lag 20 were performed to assess the similarity of the monthly proportion of rotavirus infection and all types of childhood malnutrition to other meteorological indicators (mean temperature, rainfall, sea level pressure and humidity) (S3 Fig). There was a known strong seasonality in rotavirus infection [23,32, 33] and thus the seasonal Auto-regressive Integrated Moving Average (ARIMA) was finally used. A seasonal ARIMA (p, d, q) (P, D, Q) ^s^ model was employed where

- p and P are the order of the auto regressive and seasonal autoregressive terms, respectively,
- d and Dare the order of non-seasonal and seasonal differencing,
- q and Q are the order of the moving average and seasonal moving average terms and
- s = 12 represented the length of the seasonal period in months.

All explanatory variables (main exposures; monthly proportion of underweight, stunting and wasting and four meteorological indicators were first centred by calculating the deviation from its mean and these deviations were used as the predictor variables. A series of multivariable models were employed. The initial models are: Model1: adjusted for mean temperature, rainfall, sea level pressure and humidity; Model 2: Model1 with year’s stratum added (two decades: 1993–2002 and 2003–2012). Considering a sharp change in prevalence of rotavirus infection and all the indicators of malnutrition after 2000, the years of observation were categorized into two stratums which is subsequently referred to as years stratum. Model3: Model2 with added socio-demographic characteristics (monthly mean age of under 5 children, proportion of female children, use non-sanitary toilet, non-slum residence, more than one under 5 year children in the household). Next, a series of interaction models were fitted with the aim to determine variability of association between rotavirus infection and childhood malnutrition. Model 4: Model 3 plus interaction between underweight and year stratum; Model 5: Model 3 plus interaction between underweight and mean temperature; Model 6: Model 3 plus the interaction between underweight and mean rainfall; Model 7: Model 3 plus interaction between underweight and mean sea level pressure; Model 8: Model 3 plus interaction between underweight and mean humidity; Model 9: Model 3 plus interaction between underweight, mean temperature and rainfall etc. (details in S4, S5 and S6 Tables)).

To assess the possibility of a lag effect of malnutrition on proportion of rotavirus infection (adjusted for different meteorological indicators lag of one period) was also putatively fitted. AIC and BIC values were calculated to determine whether including or excluding of lag improved the model fit (S2, S4, S5 and S6 Tables). Analyses were performed separately for all three components of malnutrition (underweight, stunting and wasting).

Additionally, the yearly mean distribution of each variable was also estimated using a moving average that used one lagged term, current value and lead term. A Spearman correlation [34] was calculated to estimate the Chi-squared for trend (S1 Table).

All analyses were undertaken using STATA version13 (StataCorp, College Station, TX, USA).

## Results

**Fig 1:**
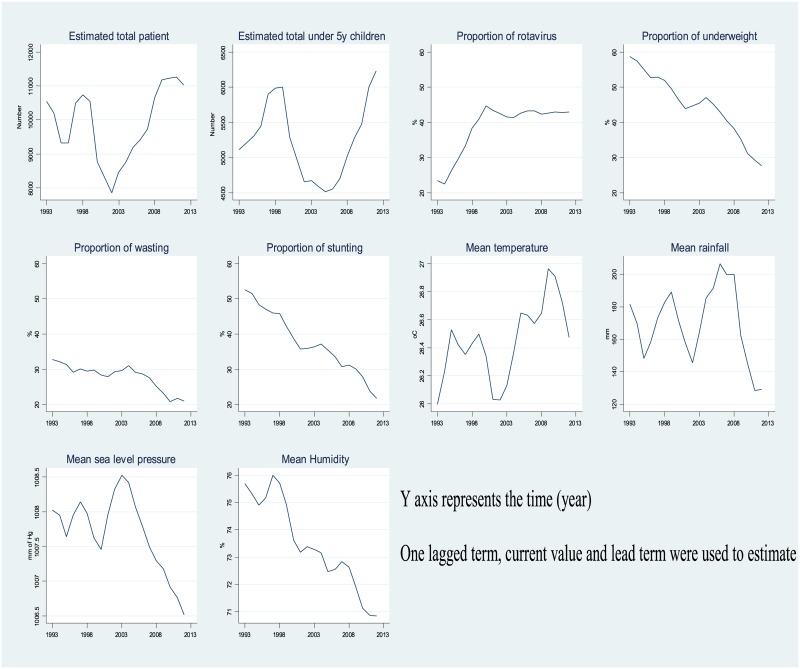
Moving average of yearly estimated to total patients and under-5 years children, proportion of rotavirus, underweight, stunting, wasting, mean temperature, rainfall, sea level pressure and humidity (1993–2012).

The yearly estimated number of under 5 years children with diarrhoea admitted to the hospital in 1993 was 5,206 and that increased to 6,892 in 2012. Similarly, the proportion of rotavirus cases during this period gradually increased from 20% in 1993 to 43% in 2012 (Chi-square for trend: p = 0.010) (Fig 1). An abrupt increase in rotavirus infection was observed in 1999 and that remained for the rest of the period.

In contrast, the proportion of children who were underweight, stunted or wasted decreased from 1993 to 2012 from 58% to 29% (Chi squared for trend: p<0.001), 53% to 21% (p<0.001) and 32% to 22% (p = <0.001) respectively (Fig 1). During this time, the mean temperature increased from 25.76°C to 26.61°C (p = 0.07) while the mean rainfall, sea level pressure and humidity decreased from 234.92 mm to 111.75 mm (p = 0.5), 1008.30 mm of hg to 1006.61 mm of hg (p = 0.02) and 76.63% to 70.26% (p<0.001) respectively.

In the unadjusted ARIMA model (Table 1), there is significant association between monthly proportion of rotavirus infection and underweight. A decrease in one unit of the monthly proportion of underweight coincided with a 0.26 increase in the monthly proportion of rotavirus infection [(95% CI: -0.43, -0.094) p = 0.002]. The association remained when adjusted for monthly mean temperature, rainfall, sea level pressure and humidity (Table 1; model 1). When adjusted for years stratum, the effect of monthly change in proportion of underweight decreased to -0.24 (95% CI: -0.42, -0.06) (Table 1, model 2). Moreover, in the final model (Table 1, model 3) adjusted for other socio-demographic factors, it accounted for a-0.19 (95% CI:-0.376, -0.003). A similar association was also observed for wasting in which one unit decrease in monthly proportion of wasting resulted in an increase of the monthly proportion of rotavirus infection by -0.27 (95% CI: -0.455, -0.075) after adjusting for all the co-variates including meteorological indicators. However, there was no association with stunting (full model for underweight, stunting and wasting are describe in S3 Table).

**Table 1:** Association between the monthly proportion of rotavirus infection and underweight, stunting, wasting for under-5 children in urban Dhaka, Bangladesh. Adjusted and unadjusted seasonal ARIMA models were fitted to the proportion of children with rotavirus infection. Covariates are outlined in the model definitions below.

	Underweight				Stunting				Wasting			
	Coef.	95% CI		p	Coef.	95% CI		p	Coef.	95% CI		p
		LL	UL			LL	UL			LL	UL	
Unadjusted	-0.260	-0.426	-0.094	0.002	-0.134	-0.288	0.020	0.089	-0.296	-0.475	-0.117	0.001
Model 1	-0.264	-0.440	-0.089	0.003	-0.134	-0.290	0.021	0.090	-0.301	-0.487	-0.115	0.002
Model 2	-0.239	-0.417	-0.062	0.008	-0.122	-0.281	0.036	0.131	-0.288	-0.476	-0.099	0.003
Model 3	-0.189	-0.376	-0.003	0.047	-0.070	-0.249	0.109	0.441	-0.265	-0.455	-0.075	0.006

Outcome: Proportion of rotavirus infection; main exposure: proportion of underweight/stunting/wasting

**Model 1**: Unadjusted+ mean centred monthly temperature, rainfall, sea level pressure, humidity

**Model 2**: Model 1 + year strata (1993–2002 vs. 2003–2012)

**Model 3**: Model 2 + mean age, proportion female, use non-sanitary toilet, non-slum residence, more than one under 5 year children in the household

Note: All estimates were in monthly basis; Centre value of underweight, mean temperature, rainfall, sea level pressure, humidity were used

When interactions were modelled, the association between monthly rotavirus infection and underweight remained significant with a variation in effect size as expected and effect size ranged between -0.14 to -0.26 with an additional variability which ranged between 0.7% to 1.52% in rotavirus infection with unadjusted model by adding different interaction terms (see S4 Table). For wasting, the effect size ranged between -0.17 and -0.36 with a variability of 0.90% to 1.14% with unadjusted model (S6 Table). Details of interaction modeling for stunting are provided in S5 Table.

## Discussion

Findings of the present study indicate a striking inverse relationship of wasting (acute malnutrition) and underweight (mixed acute and chronic malnutrition) with childhood rotavirus infection after controlling for different climate, socio-demographic and sanitation practices among under-5 years children in Dhaka. However, while a similar association with stunting (chronic malnutrition) was also observed it was not statistically significant. This nutrition interaction in which rotavirus disproportionately affects better nourished children, has been previously described [35]. Notably, this is in contrast to childhood malnutrition as an established risk factor for diarrhoeal disease due to most other enteric pathogens [36].

It is evident that rotavirus infection is associated with the variability of climate factors [22] with strong seasonal increases especially during cooler and dries months [23,32,33]. A one degree centigrade increase of temperature above a threshold (29 degrees centigrade) was associated with a 40% increase in rotavirus diarrhoea and there was a linear inverse relationship between the number of cases of rotavirus diarrhoea with relative humidity [22]. To our knowledge, this is the only study that estimated the effect of changing childhood malnutrition and its association with rotavirus infection after controlling for the ambient temperature, rainfall, sea level pressure and humidity (Model 1). These analyses indicate that not only climatic factors but also improved nutrition or over nutrition might have a significant role in the pathogenesis of rotavirus infection [12–14]. However, other man-made influences including unplanned urbanization with rapid land coverage and reduced green spaces in the Dhaka megacity and improper sewerage might be additional contributors to the causal path [5–7,37,38]. After adjustment for host (age and sex) and demographic (residence and under 5 children in the household) characteristics and sanitation practices, the significant association indicates a strong relationship between rotavirus infection and acute and chronic malnutrition.

Rotavirus, a major pathogens responsible for acute watery diarrhoea, perhaps requires healthy intestinal epithelium for attachment and pathogenesis [14]. In animal models, malnutrition results in a decreased number of cells and impaired epithelial proliferation in the small intestinal mucosa [12] that inhibit cellular attachment by the virus [13]. In addition to natural infection, it is plausible that a predilection of rotavirus for healthy, nutritionally intact intestinal epithelium is a factor in the observed immune response to Rotarix oral vaccine in which meant seroconversion rates to Rotarix were 86%, 75%, and 63% in high, middle, and low income countries respectively [39].

However, immune-compromised, severely malnourished children [11] are more prone to have infection rotavirus [16]. A reduction of acute malnutrition and, increases in the proportion of well-nourished children, as well as the recent trend towards an increasing prevalence of childhood obesity among under-5 children at DSSS [24], might positively contributed for an acute infection [15]. On the other hand, genetic variation might predict chronic malnutrition such as stunting [40], which may not be interlinked with acute infection [41].

In the present study, we observed that in the early 1990’s (1993) the proportion of all the malnutrition subtypes (underweight, wasting and stunting) were relatively high among the under5 yeras children and the proportion of rotavirus was low as was mean ambient temperature. However, from 1993 onwards, the proportion of under 5 years children with malnutrition gradually decreased and rotavirus diarrhoea increased. During 1999–2001, there was a sudden peak of rotavirus diarrhoea associated with a decreased mean level of all of the examined climate factors, in addition to reduced malnutrition rates which might have further affected the association. It is perhaps relevant that from 2002 onwards, the proportion of children under 5 years with malnutrition, mean relative humidity, rainfall and sea level pressure all decreased steadily whereas mean ambient temperature gradually increased. We employed a series of interaction models between all three components of malnutrition and climate factors and year stratum (Model 4–13; S4, S5 and S6 Tables) and estimated the variability of associations. The association largely remained significant with some degree of variability in the effect size.

## Strengths and limitations

Considering methodologic strengths of the current study are the inclusion of long-term observations with unbiased systematic samples and the quality assessment of nutrition status by trained personnel, and quality laboratory performance with same method of detection of rotavirus over the study period. The adjusting for possible confounding socio-economic factors and performing seasonal ARIMA models with a series of likely interactions are the other strengths. However, we did not consider other potentially important factors like vegetation index, per-capita land, river water level, or land surface temperature which might influence the causal path in transition of rotavirus disease [8,9]. Hospital based data might not adequately represent the population at large and might have a bias towards patients with less severe rotavirus illness and who seek care less often at hospital facilities. Finally, patients enrolled in the DDSS were self-referred and there is no subject-specific information on of their conditions, including spatial variability.

## Conclusion

Findings of the present study indicate an inverse association between acute childhood malnutrition and rotavirus diarrhoea among a population of children less than 5 years of age in urban Dhaka, Bangladesh after adjusting for important climate, socio-demographic and sanitation factors. An insignificant inverse association; however, was observed for chronic malnutrition. The relationship between recent nutritional transition and rotavirus infection in the mega city might be additionally influence by other man-made factors. As a result, an alternative intervention other than man made factors may be warranted such as mass vaccination may be needed to prevention and control of childhood rotavirus infection.

## Supporting information

S1 Table Spearman correlation between time (year) and yearly total patient numbers, number of under-5 children admitted to Dhaka hospital, proportion of under 5 rotavirus diarrhoea, underweight, wasting, stunting, mean temperature, mean rainfall, mean sea level pressure, mean humidity, mean age, proportion of female, use non-sanitary toilet, slum residence, household had under 5 years children. (DOCX)

S2 Table Akaike information criterion and Bayesian information criterion for seasonal ARIMA models to determine best fit model. (DOCX)

S3 Table Full ARIMA model of association between monthly proportion of rotavirus infection and main exposure (underweight, stunting and wasting) and other co-varieties. (DOCX)

S4 Table Association between monthly proportion of rotavirus and underweight of seasonal ARIMA models using different integrations between underweight and climate factors (effect size, Akaike information criterion, Bayesian information criterion, R square and variability of different models with unadjusted model). (DOCX)

S5 Table Association between monthly proportion of rotavirus and stunting of seasonal ARIMA models using different integrations between stunting and climate factors (effect size, Akaike information criterion, Bayesian information criterion, R square and variability of different models with unadjusted model). (DOCX)

S6 Table Association between monthly proportion of rotavirus and wasting of seasonal ARIMA models using different integrations between wasting and climate factors (effect size, Akaike information criterion, Bayesian information criterion, R square and variability of different models with unadjusted model). (DOCX)

S1 Fig Autocorrelations of monthly proportion of rotavirus, underweight, stunting and wasting, and monthly mean temperature, rainfall, sea level pressure and humidity. (PDF)

S2 Fig Partial autocorrelations of monthly proportion of rotavirus, underweight, stunting and wasting, and monthly mean temperature, rainfall, sea level pressure and humidity. (PDF)

S3 Fig Cross-correlations of monthly proportion of rotavirus and underweight, stunting, wasting, monthly mean temperature, rainfall, sea level pressure and humidity. (PDF)
