# Supplementary material for: Long-term impact of changing childhood malnutrition on rotavirus diarrhoea: Two decades of adjusted association with climate and socio-demographic factors from urban Bangladesh
Source: PLoS One. 2017 Sep 6;12(9):e0179418. doi: 10.1371/journal.pone.0179418 (PMC5587254; doi:10.1371/journal.pone.0179418)
Supplement: S1 Table — (DOCX) [file pone.0179418.s001.docx]

**S1 Table**: Spearman correlation between time (year) and yearly total patient numbers, number of under-5 children admitted to Dhaka hospital, proportion of under 5 rotavirus diarrhoea, underweight, wasting, stunting, mean temperature, mean rainfall, mean sea level pressure, mean humidity, mean age, proportion of female, use non-sanitary toilet, slum residence, household had under 5 years children

|  | Year | Total patient | Total Under-5 children | % rotavirus | % underweight | % stunting | % wasting | Mean temperature | Mean rainfall | Mean sea level pressure | Mean humidity | Mean age | % Female | % use non-sanitary toilet | % slum residence | % houshole had u5 >1 |
| --- | --- | --- | --- | --- | --- | --- | --- | --- | --- | --- | --- | --- | --- | --- | --- | --- |
| Year | 1 |  |  |  |  |  |  |  |  |  |  |  |  |  |  |  |
| Total patient | 0.277 | 1 |  |  |  |  |  |  |  |  |  |  |  |  |  |  |
|  | - |  |  |  |  |  |  |  |  |  |  |  |  |  |  |  |
| Total Under-5 children | -0.008 | 0.666 | 1 |  |  |  |  |  |  |  |  |  |  |  |  |  |
|  | - | 0.001 |  |  |  |  |  |  |  |  |  |  |  |  |  |  |
| % rotavirus | 0.564 | -0.178 | -0.013 | 1 |  |  |  |  |  |  |  |  |  |  |  |  |
|  | 0.010 | - | - |  |  |  |  |  |  |  |  |  |  |  |  |  |
| % underweight | -0.941 | -0.163 | -0.039 | -0.674 | 1 |  |  |  |  |  |  |  |  |  |  |  |
|  | <0.001 | - | - | 0.001 |  |  |  |  |  |  |  |  |  |  |  |  |
| % stunting | -0.944 | -0.179 | -0.028 | -0.647 | 0.977 | 1 |  |  |  |  |  |  |  |  |  |  |
|  | <0.001 | - | - | 0.002 | <0.001 |  |  |  |  |  |  |  |  |  |  |  |
| % wasting | -0.723 | -0.196 | -0.227 | -0.665 | 0.856 | 0.779 | 1 |  |  |  |  |  |  |  |  |  |
|  | 0.000 | - | - | 0.001 | <0.001 | 0.000 |  |  |  |  |  |  |  |  |  |  |
| Mean temperature | 0.418 | 0.349 | 0.211 | 0.269 | -0.337 | -0.334 | -0.323 | 1 |  |  |  |  |  |  |  |  |
|  | - | - | - | - | - | - | - |  |  |  |  |  |  |  |  |  |
| Mean rainfall | -0.144 | 0.098 | -0.193 | -0.042 | 0.253 | 0.196 | 0.253 | -0.050 | 1 |  |  |  |  |  |  |  |
|  | - | - | - | - | - | - | - | - |  |  |  |  |  |  |  |  |
| Mean sea level pressure | -0.517 | -0.449 | -0.475 | -0.478 | 0.593 | 0.594 | 0.612 | -0.579 | 0.083 | 1 |  |  |  |  |  |  |
|  | 0.020 | 0.047 | 0.035 | 0.033 | 0.006 | 0.006 | 0.004 | 0.008 | - |  |  |  |  |  |  |  |
| Mean humidity | -0.890 | -0.115 | 0.095 | -0.608 | 0.866 | 0.845 | 0.702 | -0.462 | 0.265 | 0.505 | 1 |  |  |  |  |  |
|  | <0.001 | - | - | 0.005 | <0.001 | <0.001 | 0.001 | 0.041 | - | 0.023 |  |  |  |  |  |  |
| Mean age | -0.352 | 0.215 | -0.204 | -0.710 | 0.558 | 0.549 | 0.543 | -0.093 | 0.316 | 0.426 | 0.314 | 1 |  |  |  |  |
|  |  |  |  | 0.001 | 0.011 | 0.012 | 0.013 | - | - | - | - |  |  |  |  |  |
| % Female | -0.353 | -0.317 | -0.182 | 0.104 | 0.259 | 0.328 | 0.060 | -0.251 | 0.320 | 0.173 | 0.302 | -0.236 | 1 |  |  |  |
|  | - | - | - | - | - | - | - | - | - | - | - | - |  |  |  |  |
| % use non-sanitary toilet | 0.445 | 0.612 | 0.405 | 0.123 | -0.475 | -0.454 | -0.445 | 0.200 | -0.156 | -0.713 | -0.423 | -0.221 | 0.008 | 1 |  |  |
|  | 0.049 | 0.004 | 0.076 | - | 0.034 | 0.044 | 0.049 | - | - | 0.000 | - | - | - |  |  |  |
| % slum residence | 0.917 | 0.205 | -0.039 | 0.517 | -0.931 | -0.931 | -0.750 | 0.275 | -0.104 | -0.582 | -0.848 | -0.381 | -0.284 | 0.514 | 1 |  |
|  | <0.001 | - | - | 0.020 | <0.001 | <0.001 | 0.000 |  |  | 0.007 | <0.001 | 0.098 |  | 0.020 |  |  |
| % household had u5 >1 | -0.669 | -0.478 | 0.105 | -0.227 | 0.599 | 0.623 | 0.444 | -0.358 | -0.196 | 0.459 | 0.621 | -0.074 | 0.301 | -0.492 | -0.734 | 1 |
|  | 0.001 | - | - | - | 0.005 | 0.003 | - | - | - | 0.042 | 0.004 | - | - | 0.028 | 0.000 |  |

Only significant at 5% level were presented
