# Supplementary material for: Long-term impact of changing childhood malnutrition on rotavirus diarrhoea: Two decades of adjusted association with climate and socio-demographic factors from urban Bangladesh
Source: PLoS One. 2017 Sep 6;12(9):e0179418. doi: 10.1371/journal.pone.0179418 (PMC5587254; doi:10.1371/journal.pone.0179418)
Supplement: S2 Table — (DOCX) [file pone.0179418.s002.docx]

**S2 Table**: Akaike information criterion and Bayesian information criterion for seasonal ARIMA models to determine best fit model

|  | **Underweight** | | **Stunting** | | **Wasting** | |
| --- | --- | --- | --- | --- | --- | --- |
| (p, d, q) (P, D, Q)s | **AIC** | **BIC** | **AIC** | **BIC** | **AIC** | **BIC** |
| (1,0,1) (1,0,1)_12_ | 1659.55 | 1683.92 | 1668.58 | 1692.94 | 1660.56 | 1684.92 |
| (0,1,1) (0,1,1)_12_ | 1587.60 | 1604.72 | 1595.73 | 1612.86 | 1582.06 | 1599.18 |
| (1,1,0) (1,1,0)_12_ | 1662.28 | 1679.40 | 1666.86 | 1683.98 | 1658.27 | 1675.40 |
| **(1,1,1) (1,1,1)_12_** | **1553.94** | **1577.91** | **1561.45** | **1585.43** | **1551.91** | **1575.88** |
| (2,1,1) (1,0,1)_12_ | 1642.71 | 1670.53 | 1651.06 | 1678.87 | 1640.40 | 1668.21 |
| (2,1,1) (1,1,1)_12_ | 1555.71 | 1583.11 | 1563.20 | 1590.60 | 1553.81 | 1581.21 |
| (2,2,2) (1,1,1)_12_ | 1602.46 | 1633.24 | 1604.51 | 1631.88 | 1598.71 | 1626.08 |
| ▪ p and P- the auto regressive and seasonal autoregressive, respectively; ▪ d and D- the non-seasonal differences and seasonal differencing, respectively; ▪ q and Q- were the moving average parameters and seasonal moving average parameters, respectively. ▪ s- represented the length of the seasonal period. | | | | | | |

***Bold*** *indicated best fit model*
