# Supplementary material for: Long-term impact of changing childhood malnutrition on rotavirus diarrhoea: Two decades of adjusted association with climate and socio-demographic factors from urban Bangladesh
Source: PLoS One. 2017 Sep 6;12(9):e0179418. doi: 10.1371/journal.pone.0179418 (PMC5587254; doi:10.1371/journal.pone.0179418)
Supplement: S3 Table — (DOCX) [file pone.0179418.s003.docx]

**S3 Table**: Full ARIMA model of association between monthly proportion of rotavirus infection and main exposure (underweight, stunting and wasting) and other co-varieties

|  | A | | | |  | B | | | |  | B | | | |
| --- | --- | --- | --- | --- | --- | --- | --- | --- | --- | --- | --- | --- | --- | --- |
|  |  | 95% CI | |  |  |  | 95% CI | |  |  |  | 95% CI | |  |
|  | Coef. | LL | UL | p |  | Coef. | LL | UL | p |  | Coef. | LL | UL | p |
| Underweight ^A^/ stunting ^B^/ wasting ^C^ | -0.189 | -0.376 | -0.003 | 0.047 |  | -0.070 | -0.249 | 0.109 | 0.441 |  | -0.265 | -0.455 | -0.075 | 0.006 |
| Mean temperature | 0.042 | -1.394 | 1.478 | 0.954 |  | 0.115 | -1.361 | 1.591 | 0.879 |  | 0.210 | -1.234 | 1.655 | 0.775 |
| Mean rainfall | 0.002 | -0.008 | 0.012 | 0.729 |  | 0.002 | -0.007 | 0.012 | 0.634 |  | 0.002 | -0.007 | 0.012 | 0.648 |
| Mean sea level pressure | 0.077 | -0.841 | 0.995 | 0.869 |  | 0.208 | -0.714 | 1.130 | 0.658 |  | 0.171 | -0.720 | 1.063 | 0.707 |
| Mean humidity | 0.028 | -0.298 | 0.354 | 0.866 |  | 0.007 | -0.318 | 0.331 | 0.967 |  | 0.045 | -0.270 | 0.361 | 0.778 |
| Year stratum | -3.156 | -10.230 | 3.917 | 0.382 |  | -4.267 | -11.301 | 2.767 | 0.234 |  | -3.164 | -10.095 | 3.766 | 0.371 |
|  | -8.985 | -18.769 | 0.798 | 0.072 |  | -9.878 | -19.810 | 0.055 | 0.051 |  | -9.082 | -18.879 | 0.715 | 0.069 |
| Proportion of female children | -0.116 | -0.299 | 0.066 | 0.211 |  | -0.133 | -0.315 | 0.050 | 0.155 |  | -0.125 | -0.302 | 0.051 | 0.164 |
| Proportion of use of non-sanitary toilet | -0.062 | -0.730 | 0.606 | 0.856 |  | -0.046 | -0.726 | 0.634 | 0.895 |  | 0.078 | -0.561 | 0.718 | 0.810 |
| Proportion of non-slum resident | 0.053 | -0.256 | 0.361 | 0.738 |  | 0.108 | -0.190 | 0.407 | 0.476 |  | 0.112 | -0.192 | 0.416 | 0.472 |
| Proportion household had > 1 under 5 children | 0.212 | -0.002 | 0.426 | 0.053 |  | 0.211 | -0.003 | 0.424 | 0.054 |  | 0.217 | 0.011 | 0.422 | 0.039 |

Outcome: monthly proportion of rotavirus infection.

Coef.: Coefficient; CI: Confidence interval; LL: Lower limit of CI; UL: Upper limit of CI; p: probability
