# Supplementary material for: Long-term impact of changing childhood malnutrition on rotavirus diarrhoea: Two decades of adjusted association with climate and socio-demographic factors from urban Bangladesh
Source: PLoS One. 2017 Sep 6;12(9):e0179418. doi: 10.1371/journal.pone.0179418 (PMC5587254; doi:10.1371/journal.pone.0179418)
Supplement: S4 Table — (DOCX) [file pone.0179418.s004.docx]

**S4 Table**: Association between monthly proportion of rotavirus and underweight of seasonal ARIMA models using different integrations between underweight and climate factors (effect size, Akaike information criterion, Bayesian information criterion, R square and variability of different models with unadjusted model)

|  | Effect size of main exposure | | | |  | Effect size of interaction | | | |  | Model estimates | |  |  | LR test | |  |
| --- | --- | --- | --- | --- | --- | --- | --- | --- | --- | --- | --- | --- | --- | --- | --- | --- | --- |
|  |  | 95% CI | |  |  |  | 95% CI | |  |  |  |  |  | R square |  |  |  |
|  | Coef. | LL | UL | p |  | Coef. | LL | UL | p |  | AIC | BIC |  |  | Chi square | P | Variability |
| Unadjusted | -0.260 | -0.426 | -0.094 | 0.002 |  | - | - | - | - |  | 1553.94 | 1577.91 |  | 0.0254 | - | - | - |
| Model 1 | -0.264 | -0.440 | -0.089 | 0.003 |  | - | - | - | - |  | 1561.32 | 1599.00 |  | 0.0255 | 0.62 | 0.9613 | 0.00005 |
| Model 2 | -0.239 | -0.417 | -0.062 | 0.008 |  | - | - | - | - |  | 1561.17 | 1602.27 |  | 0.0268 | 2.77 | 0.7356 | 0.00139 |
| Model 3 | -0.189 | -0.376 | -0.003 | 0.047 |  | - | - | - | - |  | 1558.75 | 1616.97 |  | 0.0149 | 15.19 | 0.1252 | -0.01049 |
| Model 4 | -0.145 | -0.409 | 0.118 | 0.280 |  | -0.068 | -0.381 | 0.245 | 0.671 |  | 1560.50 | 1622.15 |  | 0.0135 | 15.44 | 0.1633 | -0.01195 |
| Model 5 | -0.189 | -0.375 | -0.003 | 0.046 |  | 0.001 | -0.034 | 0.035 | 0.968 |  | 1558.74 | 1616.97 |  | 0.0148 | 15.2 | 0.1251 | -0.01063 |
| Model 6 | -0.189 | -0.369 | -0.010 | 0.039 |  | 0.001 | 0.000 | 0.001 | 0.014 |  | 1553.45 | 1615.10 |  | 0.0107 | 22.49 | 0.0209 | -0.01470 |
| Model 7 | -0.189 | -0.375 | -0.003 | 0.047 |  | -0.008 | -0.031 | 0.015 | 0.494 |  | 1560.35 | 1622.00 |  | 0.0143 | 15.59 | 0.1569 | -0.01110 |
| Model 8 | -0.181 | -0.365 | 0.004 | 0.055 |  | 0.015 | 0.000 | 0.030 | 0.054 |  | 1553.87 | 1612.10 |  | 0.0103 | 20.07 | 0.0286 | -0.01515 |
| Model 9 | -0.264 | -0.477 | -0.051 | 0.015 |  | 0.000 | 0.000 | 0.000 | 0.131 |  | 1557.44 | 1619.09 |  | 0.0199 | 18.5 | 0.0707 | -0.00551 |
| Model 10 | -0.213 | -0.409 | -0.017 | 0.033 |  | 0.003 | -0.002 | 0.008 | 0.321 |  | 1559.12 | 1620.77 |  | 0.0182 | 16.82 | 0.1132 | -0.00727 |
| Model 11 | -0.218 | -0.426 | -0.010 | 0.040 |  | 0.000 | 0.000 | 0.000 | 0.550 |  | 1560.18 | 1621.83 |  | 0.0163 | 15.76 | 0.1502 | -0.00916 |
| Model 12 | -0.198 | -0.386 | -0.010 | 0.039 |  | 0.000 | 0.000 | 0.000 | 0.502 |  | 1558.05 | 1616.27 |  | 0.0135 | 15.89 | 0.1028 | -0.01198 |
| Model 13 | -0.226 | -0.423 | -0.028 | 0.025 |  | 0.000 | 0.000 | 0.000 | 0.263 |  | 1559.08 | 1620.73 |  | 0.0184 | 16.86 | 0.112 | -0.00708 |

Outcome: Proportion of rotavirus infection; main exposure: proportion of underweight (centred)

**Model 1**: Unadjusted+ mean centred monthly temperature, rainfall, sea level pressure, humidity

**Model 2**: Model 1 + year strata (1993-2002 vs. 2003-2012)

**Model 3**: Model 2 + mean age, proportion female, use non-sanitary toilet, non-slum residence, more than one under 5 year children in the household

**Model 4**: Model 3 + Interaction between proportion of underweight and year strata

**Model 5**: Model 3 + Interaction between proportion of underweight and mean temperature

**Model 6:** Model 3 + Interaction between proportion of underweight and mean rainfall

**Model 7:** Model 3 + Interaction between proportion of underweight and mean sea level pressure

**Model 8:** Model 3 + Interaction between proportion of underweight and mean humidity

**Model 9:** Model 3 + Interaction between proportion of underweight, mean temperature and mean rainfall

**Model 10:** Model 3 + Interaction between proportion of underweight, mean temperature and mean humidity

**Model 11:** Model 3 + Interaction between proportion of underweight, mean rainfall and mean humidity

**Model 12:** Model 3 + Interaction between proportion of underweight, mean temperature, mean rainfall, mean humidity

**Model 13:** Model 3 + Interaction between proportion of underweight, mean temperature, mean rainfall, mean sea level pressure and mean humidity

*Note: All estimates were in monthly basis; Centred value of underweight, mean temperature, rainfall, sea level pressure, humidity were used.* Coef.: Coefficient; CI: Confidence interval; LL: Lower limit of CI; UL: Upper limit of CI; p: probability; LR: Likelihood ratio

*alue of underweight, mean temperature, rainfall, sea level pressure, humidity were used.* Coef.: Coefficient; CI: Confidence interval; LL: Lower limit of CI; UL: Upper limit of CI; p: probability; LR: Likelihood ratio
