# Supplementary material for: Long-term impact of changing childhood malnutrition on rotavirus diarrhoea: Two decades of adjusted association with climate and socio-demographic factors from urban Bangladesh
Source: PLoS One. 2017 Sep 6;12(9):e0179418. doi: 10.1371/journal.pone.0179418 (PMC5587254; doi:10.1371/journal.pone.0179418)
Supplement: S5 Table — (DOCX) [file pone.0179418.s005.docx]

**S5 Table**: Association between monthly proportion of rotavirus and stunting of seasonal ARIMA models using different integrations between stunting and climate factors (effect size, Akaike information criterion, Bayesian information criterion, R square and variability of different models with unadjusted model).

|  | Effect size of main exposure | | | |  | Effect size of interaction | | | |  | Model estimates | |  |  | LR test | |  |
| --- | --- | --- | --- | --- | --- | --- | --- | --- | --- | --- | --- | --- | --- | --- | --- | --- | --- |
|  |  | 95% CI | |  |  |  | 95% CI | |  |  |  |  |  | R square |  |  |  |
|  | Coef. | LL | UL | p |  | Coef. | LL | UL | p |  | AIC | BIC |  |  | Chi square | P | Variability |
| Unadjusted | -0.134 | -0.288 | 0.020 | 0.089 |  | - | - | - | - |  | 1561.45 | 1585.43 |  | 0.0305 | - | - | - |
| Model 1 | -0.134 | -0.290 | 0.021 | 0.090 |  | - | - | - | - |  | 1569.04 | 1606.72 |  | 0.0298 | 0.41 | 0.9817 | -0.00065 |
| Model 2 | -0.122 | -0.281 | 0.036 | 0.131 |  | - | - | - | - |  | 1567.45 | 1608.54 |  | 0.0301 | 4.01 | 0.5483 | -0.00044 |
| Model 3 | -0.070 | -0.249 | 0.109 | 0.441 |  | - | - | - | - |  | 563.24 | 1621.47 |  | 0.0162 | 18.21 | 0.0515 | -0.01428 |
| Model 4 | -0.037 | -0.279 | 0.205 | 0.765 |  | -0.063 | -0.399 | 0.272 | 0.711 |  | 1565.06 | 1626.70 |  | 0.0152 | 18.4 | 0.0728 | -0.01528 |
| Model 5 | -0.059 | -0.236 | 0.119 | 0.517 |  | 0.022 | -0.011 | 0.054 | 0.190 |  | 1561.17 | 1619.40 |  | 0.0106 | 20.28 | 0.0267 | -0.01994 |
| Model 6 | -0.055 | -0.227 | 0.118 | 0.535 |  | 0.001 | 0.000 | 0.002 | 0.003 |  | 1553.96 | 1612.18 |  | 0.0100 | 27.5 | 0.0022 | -0.02048 |
| Model 7 | -0.052 | -0.229 | 0.125 | 0.565 |  | -0.024 | -0.046 | -0.003 | 0.027 |  | 1558.56 | 1616.78 |  | 0.0117 | 22.89 | 0.0111 | -0.01880 |
| Model 8 | -0.065 | -0.238 | 0.109 | 0.466 |  | 0.018 | 0.003 | 0.033 | 0.017 |  | 1556.53 | 1614.76 |  | 0.0097 | 24.92 | 0.0055 | -0.02083 |
| Model 9 | -0.103 | -0.312 | 0.105 | 0.330 |  | 0.000 | 0.000 | 0.000 | 0.541 |  | 1564.77 | 1626.42 |  | 0.0187 | 18.69 | 0.067 | -0.01181 |
| Model 10 | -0.087 | -0.275 | 0.102 | 0.369 |  | 0.002 | -0.004 | 0.007 | 0.514 |  | 1564.59 | 1626.24 |  | 0.0185 | 18.86 | 0.0636 | -0.01203 |
| Model 11 | -0.097 | -0.301 | 0.107 | 0.350 |  | 0.000 | 0.000 | 0.000 | 0.493 |  | 1564.65 | 1626.29 |  | 0.0172 | 18.81 | 0.0646 | -0.01331 |
| Model 12 | -0.086 | -0.266 | 0.095 | 0.352 |  | 0.000 | 0.000 | 0.000 | 0.198 |  | 1560.84 | 1619.06 |  | 0.0129 | 20.62 | 0.0239 | -0.01759 |
| Model 13 | -0.096 | -0.285 | 0.092 | 0.317 |  | 0.000 | 0.000 | 0.000 | 0.327 |  | 1564.05 | 1625.70 |  | 0.0186 | 19.41 | 0.0542 | -0.01188 |

Outcome: Proportion of rotavirus infection; main exposure: proportion of stunting (centred)

**Model 1**: Unadjusted+ mean centred monthly temperature, rainfall, sea level pressure, humidity

**Model 2**: Model 1 + year strata (1993-2002 vs. 2003-2012)

**Model 3**: Model 2 + mean age, proportion female, use non-sanitary toilet, non-slum residence, more than one under 5 year children in the household

**Model 4**: Model 3 + Interaction between proportion of underweight and year strata

**Model 5**: Model 3 + Interaction between proportion of underweight and mean temperature

**Model 6:** Model 3 + Interaction between proportion of underweight and mean rainfall

**Model 7:** Model 3 + Interaction between proportion of underweight and mean sea level pressure

**Model 8:** Model 3 + Interaction between proportion of underweight and mean humidity

**Model 9:** Model 3 + Interaction between proportion of underweight, mean temperature and mean rainfall

**Model 10:** Model 3 + Interaction between proportion of underweight, mean temperature and mean humidity

**Model 11:** Model 3 + Interaction between proportion of underweight, mean rainfall and mean humidity

**Model 12:** Model 3 + Interaction between proportion of underweight, mean temperature, mean rainfall, mean humidity

**Model 13:** Model 3 + Interaction between proportion of underweight, mean temperature, mean rainfall, mean sea level pressure and mean humidity

*Note: All estimates were in monthly basis; Centred value of underweight, mean temperature, rainfall, sea level pressure, humidity were used.* Coef.: Coefficient; CI: Confidence interval; LL: Lower limit of CI; UL: Upper limit of CI; p: probability; LR: Likelihood ratio
