# Supplementary material for: Long-term impact of changing childhood malnutrition on rotavirus diarrhoea: Two decades of adjusted association with climate and socio-demographic factors from urban Bangladesh
Source: PLoS One. 2017 Sep 6;12(9):e0179418. doi: 10.1371/journal.pone.0179418 (PMC5587254; doi:10.1371/journal.pone.0179418)
Supplement: S6 Table — (DOCX) [file pone.0179418.s006.docx]

**S6 Table**: Association between monthly proportion of rotavirus and wasting of seasonal ARIMA models using different integrations between wasting and climate factors (effect size, Akaike information criterion, Bayesian information criterion, R square and variability of different models with unadjusted model).

|  | Effect size of main exposure | | | |  | Effect size of interaction | | | |  | Model estimates | |  |  | LR test | |  |
| --- | --- | --- | --- | --- | --- | --- | --- | --- | --- | --- | --- | --- | --- | --- | --- | --- | --- |
|  |  | 95% CI | |  |  |  | 95% CI | |  |  |  |  |  | R square |  |  |  |
|  | Coef. | LL | UL | p |  | Coef. | LL | UL | p |  | AIC | BIC |  |  | Chi square | P | Variability |
| Unadjusted | -0.296 | -0.475 | -0.117 | 0.001 |  | - | - | - | - |  | 1551.91 | 1575.88 |  | 0.0255 | - | - | - |
| Model 1 | -0.301 | -0.487 | -0.115 | 0.002 |  | - | - | - | - |  | 1559.36 | 1597.03 |  | 0.0247 | 0.55 | 0.9683 | -0.00082 |
| Model 2 | -0.288 | -0.476 | -0.099 | 0.003 |  | - | - | - | - |  | 1556.19 | 1593.87 |  | 0.0253 | 3.72 | 0.4457 | -0.00023 |
| Model 3 | -0.265 | -0.455 | -0.075 | 0.006 |  | - | - | - | - |  | 1553.88 | 1612.10 |  | 0.0145 | 18.03 | 0.0545 | -0.01102 |
| Model 4 | -0.168 | -0.425 | 0.089 | 0.199 |  | -0.162 | -0.488 | 0.163 | 0.328 |  | 1552.65 | 1610.88 |  | 0.0109 | 19.26 | 0.0371 | -0.01455 |
| Model 5 | -0.261 | -0.452 | -0.070 | 0.007 |  | -0.027 | -0.077 | 0.024 | 0.297 |  | 1554.24 | 1615.89 |  | 0.0151 | 19.67 | 0.0501 | -0.01034 |
| Model 6 | -0.273 | -0.460 | -0.086 | 0.004 |  | 0.000 | 0.000 | 0.001 | 0.295 |  | 1552.11 | 1610.33 |  | 0.0142 | 19.8 | 0.0312 | -0.01129 |
| Model 7 | -0.270 | -0.460 | -0.080 | 0.005 |  | 0.019 | -0.016 | 0.053 | 0.289 |  | 1552.45 | 1610.68 |  | 0.0140 | 19.46 | 0.0348 | -0.01150 |
| Model 8 | -0.270 | -0.462 | -0.079 | 0.006 |  | 0.008 | -0.015 | 0.031 | 0.490 |  | 1555.24 | 1616.89 |  | 0.0137 | 18.67 | 0.0674 | -0.01184 |
| Model 9 | -0.360 | -0.595 | -0.125 | 0.003 |  | 0.000 | 0.000 | 0.001 | 0.186 |  | 1552.94 | 1614.59 |  | 0.0161 | 20.96 | 0.0337 | -0.00943 |
| Model 10 | -0.305 | -0.500 | -0.109 | 0.002 |  | 0.004 | -0.002 | 0.010 | 0.231 |  | 1554.16 | 1615.81 |  | 0.0169 | 19.74 | 0.049 | -0.00859 |
| Model 11 | -0.296 | -0.505 | -0.087 | 0.006 |  | 0.000 | 0.000 | 0.000 | 0.593 |  | 1555.35 | 1617.00 |  | 0.0152 | 18.56 | 0.0695 | -0.01024 |
| Model 12 | -0.267 | -0.460 | -0.074 | 0.007 |  | 0.000 | 0.000 | 0.000 | 0.906 |  | 1555.86 | 1617.51 |  | 0.0143 | 18.05 | 0.0805 | -0.01119 |
| Model 13 | -0.281 | -0.474 | -0.089 | 0.004 |  | 0.000 | 0.000 | 0.000 | 0.615 |  | 1553.61 | 1611.839 |  | 0.0156 | 18.29 | 0.0502 | -0.00988 |

Outcome: Proportion of rotavirus infection; main exposure: proportion of wasting (centred)

**Model 1**: Unadjusted+ mean centred monthly temperature, rainfall, sea level pressure, humidity

**Model 2**: Model 1 + year strata (1993-2002 vs. 2003-2012)

**Model 3**: Model 2 + mean age, proportion female, use non-sanitary toilet, non-slum residence, more than one under 5 year children in the household

**Model 4**: Model 3 + Interaction between proportion of underweight and year strata

**Model 5**: Model 3 + Interaction between proportion of underweight and mean temperature

**Model 6:** Model 3 + Interaction between proportion of underweight and mean rainfall

**Model 7:** Model 3 + Interaction between proportion of underweight and mean sea level pressure

**Model 8:** Model 3 + Interaction between proportion of underweight and mean humidity

**Model 9:** Model 3 + Interaction between proportion of underweight, mean temperature and mean rainfall

**Model 10:** Model 3 + Interaction between proportion of underweight, mean temperature and mean humidity

**Model 11:** Model 3 + Interaction between proportion of underweight, mean rainfall and mean humidity

**Model 12:** Model 3 + Interaction between proportion of underweight, mean temperature, mean rainfall, mean humidity

**Model 13:** Model 3 + Interaction between proportion of underweight, mean temperature, mean rainfall, mean sea level pressure and mean humidity

*Note: All estimates were in monthly basis; Centred value of underweight, mean temperature, rainfall, sea level pressure, humidity were used.* Coef.: Coefficient; CI: Confidence interval; LL: Lower limit of CI; UL: Upper limit of CI; p: probability; LR: Likelihood ratio
