# Supplementary material for: Long-term impact of changing childhood malnutrition on rotavirus diarrhoea: Two decades of adjusted association with climate and socio-demographic factors from urban Bangladesh
Source: PLoS One. 2017 Sep 6;12(9):e0179418. doi: 10.1371/journal.pone.0179418 (PMC5587254; doi:10.1371/journal.pone.0179418)

S 1 Fig.: Autocorrelations of monthly proportion of rotavirus, underweight, stunting and wasting, and monthly mean temperature, rainfall, sea level pressure and

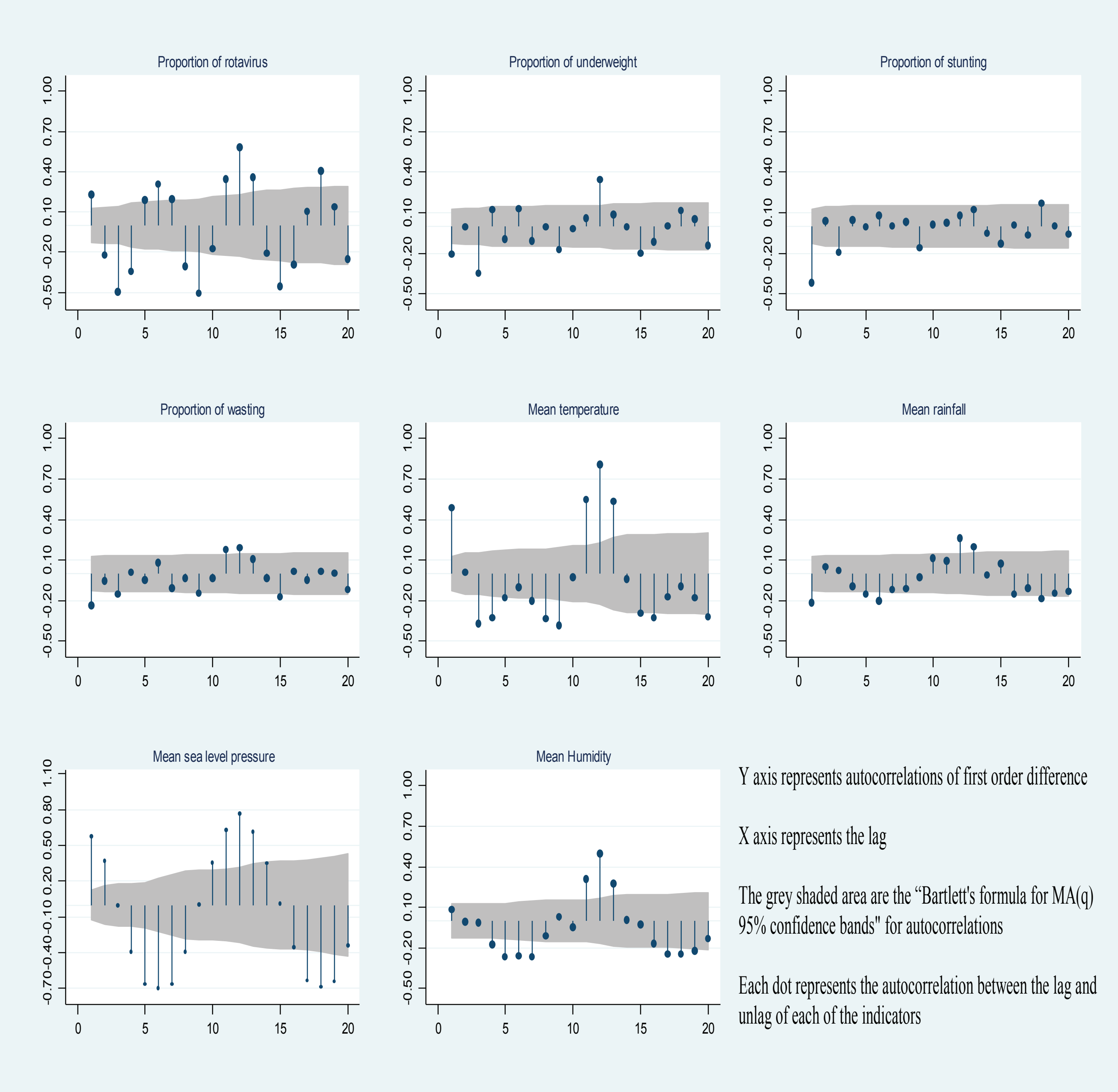

Supplement: S1 Fig — (PDF) [file pone.0179418.s007.pdf]
