# Supplementary material for: Long-term impact of changing childhood malnutrition on rotavirus diarrhoea: Two decades of adjusted association with climate and socio-demographic factors from urban Bangladesh
Source: PLoS One. 2017 Sep 6;12(9):e0179418. doi: 10.1371/journal.pone.0179418 (PMC5587254; doi:10.1371/journal.pone.0179418)
Supplement: S3 Fig — (PDF) [file pone.0179418.s009.pdf]

S 3 Fig.: Cross-correlations of monthly proportion of rotavirus and underweight, stunting, wasting, monthly mean temperature, rainfall, sea level pressure and humidity

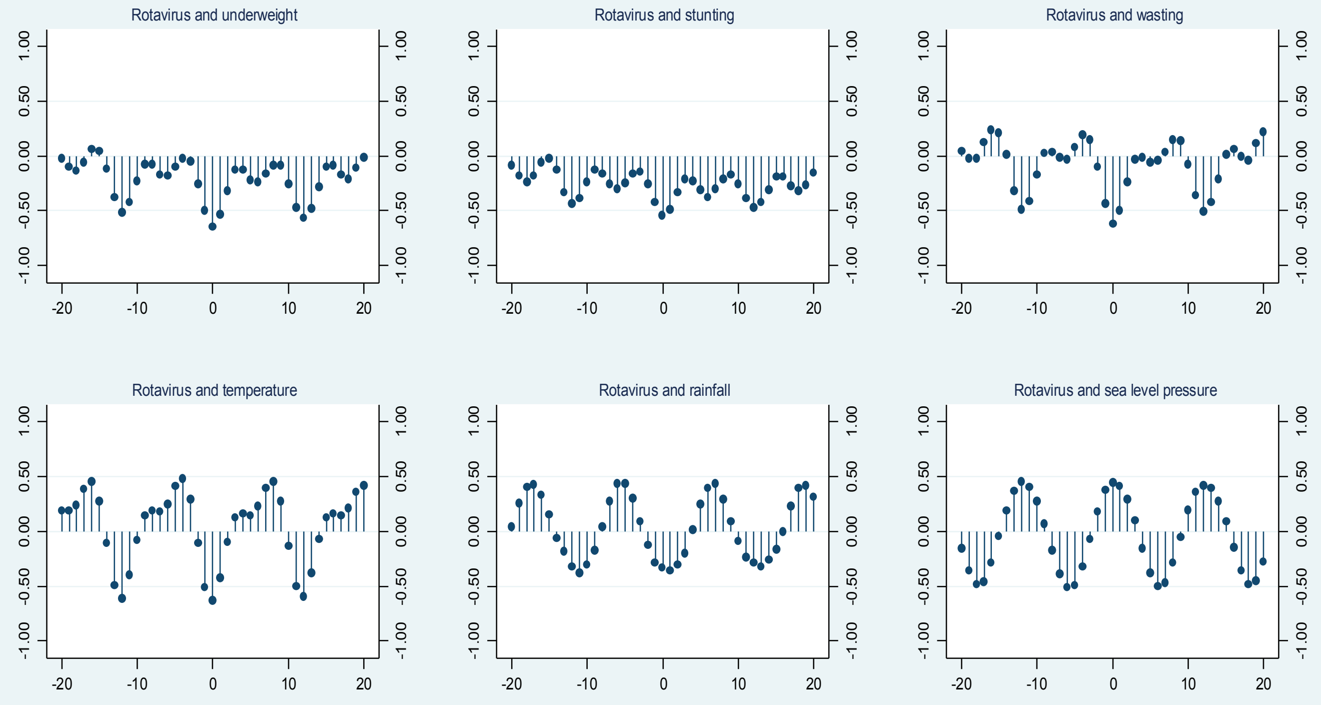

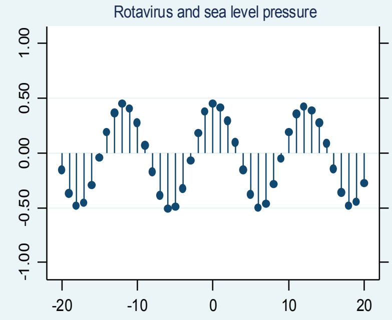

Y1 and Y2 axis represents cross-correlations of first order lag

X axis represents the lag

Each dot represents the cross-correlations between monthly proportions of rotavirus with all other individual indicators underweight, stunting, wasting, monthly mean temperature, rainfall, sea level pressure and humidity respectively
